# Whole slide image-based weakly supervised deep learning for predicting major pathological response in non-small cell lung cancer following neoadjuvant chemoimmunotherapy: a multicenter, retrospective, cohort study

**DOI:** 10.3389/fimmu.2024.1453232

**Published:** 2024-09-20

**Authors:** Dan Han, Hao Li, Xin Zheng, Shenbo Fu, Ran Wei, Qian Zhao, Chengxin Liu, Zhongtang Wang, Wei Huang, Shaoyu Hao

**Affiliations:** ^1^ Department of Radiation Oncology, Shandong University Cancer Center, Jinan, Shandong, China; ^2^ Department of Radiation Oncology, Shandong Cancer Hospital and Institute, Shandong First Medical University and Shandong Academy of Medical Sciences, Jinan, Shandong, China; ^3^ Department of Radiology, Shandong Provincial Qianfoshan Hospital, Shandong University, Jinan, Shandong, China; ^4^ Department of Radiation Oncology and Shandong Provincial Key Laboratory of Radiation Oncology, Shandong Cancer Hospital and Institute, Shandong First Medical University and Shandong Academy of Medical Sciences, Jinan, Shandong, China; ^5^ Department of Traditional Chinese Medicine, Qingdao Hospital of Traditional Chinese Medicine (Qingdao Hiser Hospital), Qingdao, China; ^6^ Department of Radiation Oncology, Shanxi Provincial Tumor Hospital, Xi’an, Shanxi, China; ^7^ Department of Radiology, Jining No.1 People’s Hospital, Jining, Shandong, China; ^8^ Department of Thoracic Surgery, Shandong University Cancer Center, Jinan, Shandong, China; ^9^ Department of Thoracic Surgery, Shandong Cancer Hospital and Institute, Shandong First Medical University, and Shandong Academy of Medical Sciences, Jinan, Shandong, China

**Keywords:** non-small cell lung cancer, major pathological response, neoadjuvant chemoimmunotherapy, whole slide image, weakly supervised learning

## Abstract

**Objective:**

Develop a predictive model utilizing weakly supervised deep learning techniques to accurately forecast major pathological response (MPR) in patients with resectable non-small cell lung cancer (NSCLC) undergoing neoadjuvant chemoimmunotherapy (NICT), by leveraging whole slide images (WSIs).

**Methods:**

This retrospective study examined pre-treatment WSIs from 186 patients with non-small cell lung cancer (NSCLC), using a weakly supervised learning framework. We employed advanced deep learning architectures, including DenseNet121, ResNet50, and Inception V3, to analyze WSIs on both micro (patch) and macro (slide) levels. The training process incorporated innovative data augmentation and normalization techniques to bolster the robustness of the models. We evaluated the performance of these models against traditional clinical predictors and integrated them with a novel pathomics signature, which was developed using multi-instance learning algorithms that facilitate feature aggregation from patch-level probability distributions.

**Results:**

Univariate and multivariable analyses confirmed histology as a statistically significant prognostic factor for MPR (*P*-value< 0.05). In patch model evaluations, DenseNet121 led in the validation set with an area under the curve (AUC) of 0.656, surpassing ResNet50 (AUC = 0.626) and Inception V3 (AUC = 0.654), and showed strong generalization in external testing (AUC = 0.611). Further evaluation through visual inspection of patch-level data integration into WSIs revealed XGBoost’s superior class differentiation and generalization, achieving the highest AUCs of 0.998 in training and robust scores of 0.818 in validation and 0.805 in testing. Integrating pathomics features with clinical data into a nomogram yielded AUC of 0.819 in validation and 0.820 in testing, enhancing discriminative accuracy. Gradient-weighted Class Activation Mapping (Grad-CAM) and feature aggregation methods notably boosted the model’s interpretability and feature modeling.

**Conclusion:**

The application of weakly supervised deep learning to WSIs offers a powerful tool for predicting MPR in NSCLC patients treated with NICT.

## Introduction

The employment of neoadjuvant chemoimmunotherapy (NICT) has risen as an effective method for managing resectable non-small cell lung cancer (NSCLC). A number of research has explored its viability and efficacy, showcasing that this strategy can enhance pathological response rates and complete tumor removal. Furthermore, it assists in managing microscopically invisible metastases, thus favorably influencing patient outcomes (1–6).

In many trials focusing on neoadjuvant immunotherapy for NSCLC, major pathological response (MPR) is considered a key predictor for overall survival (OS) and disease-free survival (DFS). However, the rates of MPR observed in current clinical research on NICT display a wide variance, ranging from 18% to 83% (1, 3–5, 7–14). This disparity underscores that not all patients derive benefit from NICT; indeed, ineffective treatment may lead to delays in surgical intervention and an increased likelihood of immune-related side effects. Consequently, crafting a dependable predictive model for MPR response to NICT in patients with resectable NSCLC is crucial, offering the potential to tailor treatments more effectively and enhance patient outcomes.

Tissue specimens stained with Hematoxylin and Eosin (H&E) contain a wealth of useful information for routine histopathological analysis. Artificial intelligence (AI) is increasingly used to analyze H&E stained histopathological images for differential diagnosis and prognosis prediction in NSCLC studies, enhancing the evaluation of conventional histological slides (15–22). This approach holds immense potential for disease research, as AI algorithms can assist clinicians and pathologists in their decision-making by analyzing whole slide images (WSIs).

Weakly supervised learning has garnered widespread attention due to its significant advantage in reducing the workload of manual annotation and has been gradually applied in the field of pathological image analysis (23–25). The classic patch-based weakly supervised method provides a specific workflow for processing histological images. Due to the expansive dimensions of WSIs, segmentation into smaller tiles is necessary for processing, with an averaging method subsequently aggregating the tile-level predictions for each slide (26). This approach has introduced a new level of flexibility and application prospects in the realm of weakly supervised learning for pathological image analysis.

In this study, we developed a weakly supervised deep learning model utilizing pre-treatment WSIs to predict MPR in patients undergoing NICT for NSCLC. The model’s predictions can serve as a reference for physicians to enhance treatment planning.

## Materials and methods

### Data collection

**Figure 1:**
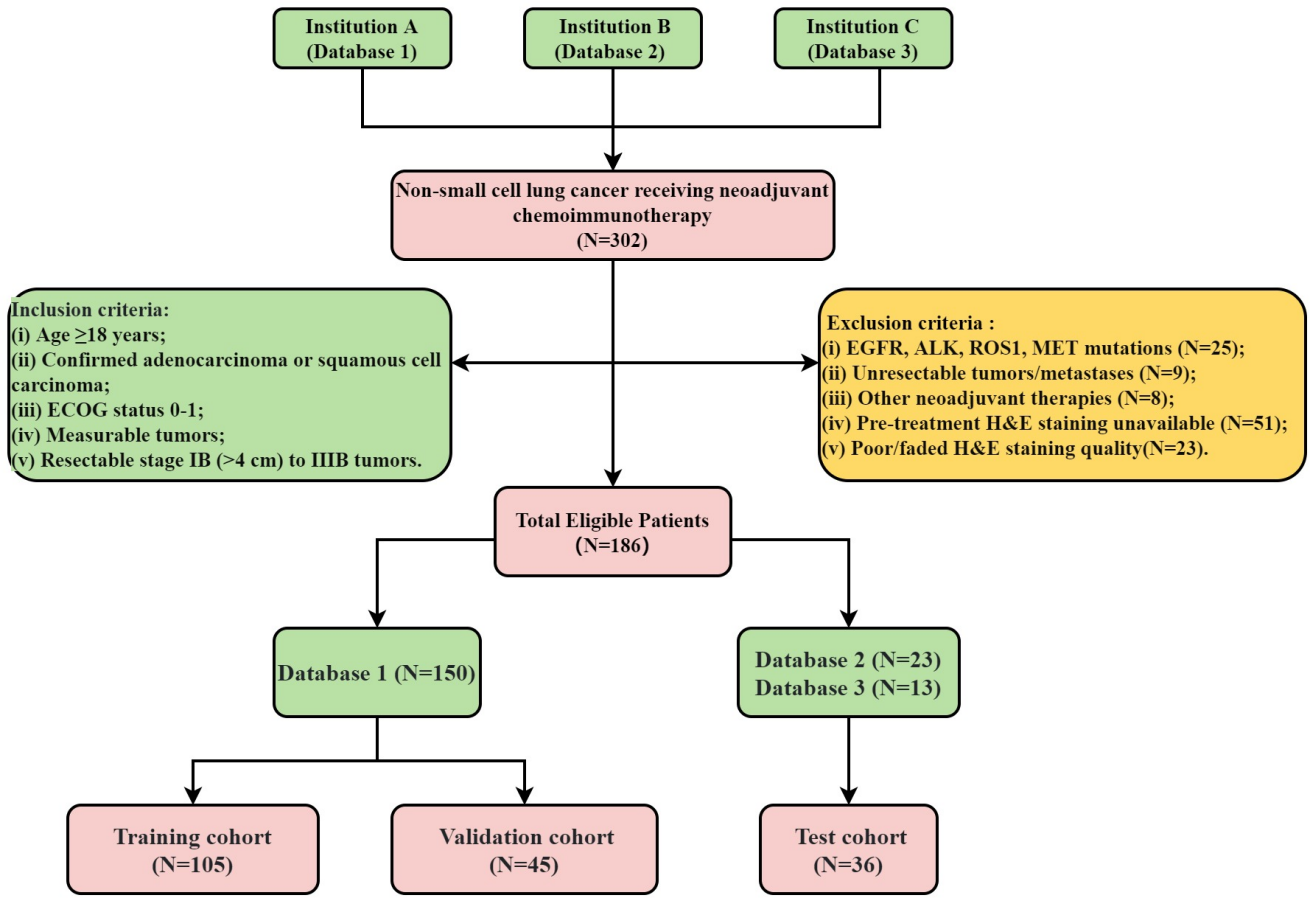
Flowchart of the cohorts used in this study.

The flowchart illustrating the cohort selection process for this study is presented in **Figure 1**. This study initially enrolled 302 patients who received NICT followed by surgical intervention from November 24, 2020, to March 10, 2024. However, 116 patients were subsequently excluded based on predefined criteria. All pre-treatment H&E-stained slides were digitized into WSIs using a WISLEAP scanner and then converted to NDPI format via NDPView2 software. Ultimately, 186 patients contributing 212 WSIs diagnosed with NSCLC were retrospectively selected from three institutions. The allocation of patients across these institutions was as follows: 150 from Shandong Cancer Hospital (Database 1), 23 from Shanxi Cancer Hospital (Database 2), and 13 from the First People’s Hospital of Jining City (Database 3). Within the training cohort, samples were apportioned into a training subset and an internal validation subset at a 7:3 ratio. Due to sample size constraints, data from Databases 2 and 3 were amalgamated to constitute the test datasets.

**Figure 2:**
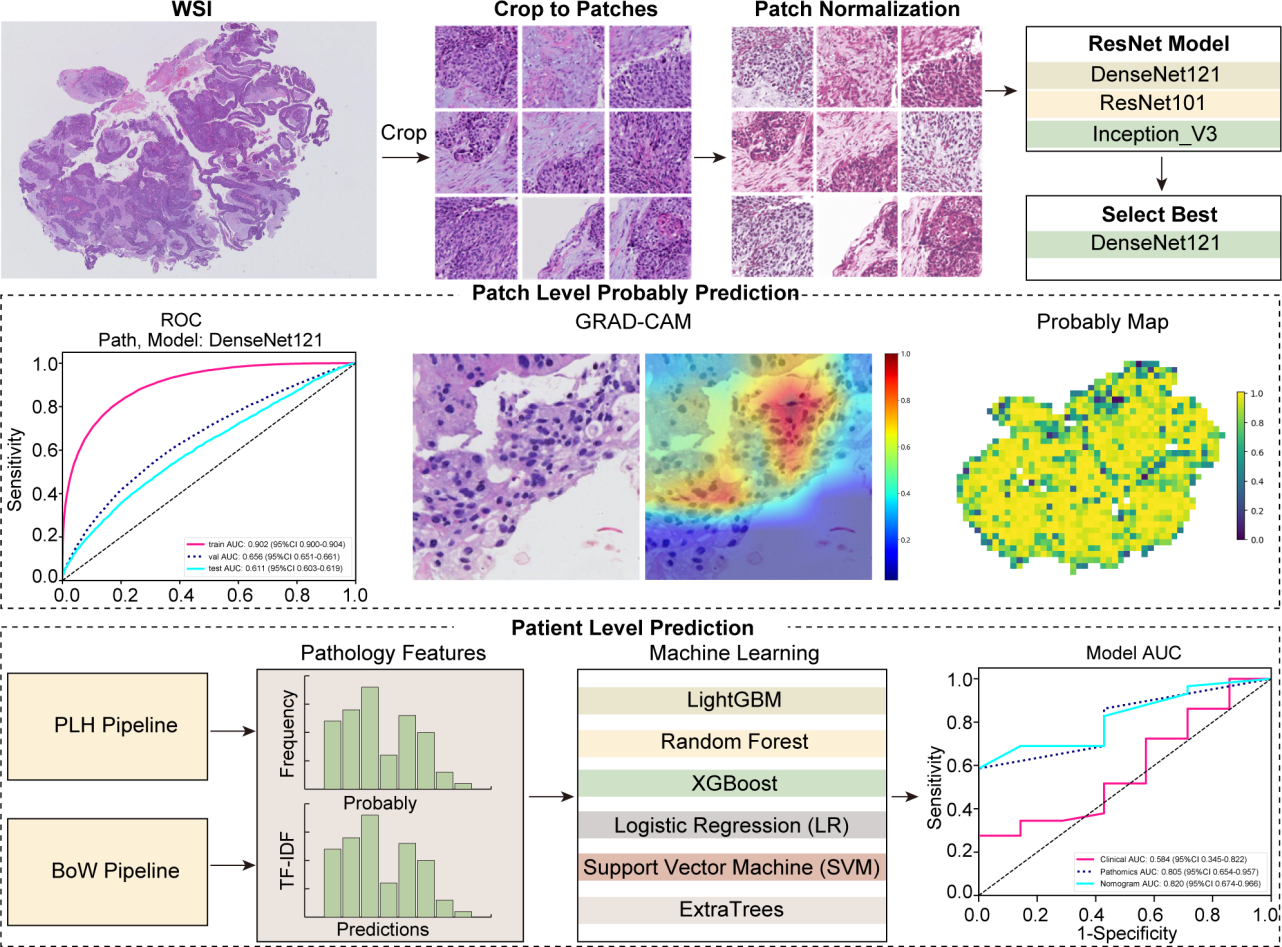
Overall workflow of the study.

Full details regarding the treatment protocols can be found in **Supplementary Data Sheet 2**. This study was conducted in accordance with the 8th edition of the American Joint Committee on Cancer (AJCC) Tumor, Node, Metastasis (TNM) staging system. MPR was defined as the presence of less than 10% viable tumor cells in the pathological examination of the surgical specimen (27). The conduct of this study was in strict compliance with the principles of the Declaration of Helsinki and received ethical clearance from the institutional review board (number: SDTHEC2024002010). This study, which was retrospectively registered with the ResearchRegistry (registration ID: researchregistry10216). Additionally, the study received further ethical approvals from the Institutional Review Board of the First People’s Hospital of Jining City (approval number: JNRM-2024-KY-037) and the Medical Ethics Committee of Shaanxi Cancer Hospital [approval number: Ethics Review No. 39 (2024)]. Owing to its retrospective design and the absence of any risk to participants, the need for informed consent was duly waived. **Figure 2** depicts the comprehensive workflow of our study.

### Data processing

In processing the WSIs, which typically span dimensions of approximately 100,000 x 50,000 pixels, we utilized a 20x magnification to capture these images, resulting in a pixel resolution of about 0.5 μm/pixel. The WSIs were subsequently divided into smaller segments of 512x512 pixels each. By employing a series of image processing techniques, including grayscale conversion, Otsu’s thresholding, and morphological operations for background removal, we efficiently eliminated all white backgrounds from these patches. This process resulted in over 17,000 distinct, non-overlapping tiles.

During the model’s training phase, we incorporated online data augmentation strategies to increase the dataset’s variability. This included random horizontal and vertical flips of the image patches. To maintain a standardized input size, we meticulously performed center cropping to adjust the dimensions to 224 x 224 pixels, and specifically to 299 x 299 pixels for the Inception V3 architecture. Additionally, Z-score normalization was applied to the RGB channels to normalize the distribution of pixel values.

### Weakly supervised learning

In our study, we employed deep learning algorithms to facilitate predictive analysis at both the micro (patch) and macro (WSI) levels. The segmentation of WSIs into smaller, discrete patches was undertaken, ensuring that each patch from a single specimen uniformly bore the same MPR designation. To predict outcomes at the patch level, we meticulously evaluated three prominent neural network architectures: DenseNet121, ResNet50, and Inception V3. The objective was to ascertain the precision with which each patch could be classified into a category mirroring its overarching WSI classification.

To improve the generalizability of our pathology model, we optimized the learning rate employing a cosine decay algorithm, ensuring a refined and effective adjustment over the training period. This approach is characterized as follows:


$$\eta_{t}=\eta_{min}^{i}+\frac{1}{2}\left(\eta_{max}^{i}-\eta_{min}^{i}\right)\left(1+\text{cos}\left(\frac{T_{cur}}{T_{i}}\pi \right)\right)$$

In this formulation, ${\text{η}}_{\text{min}}^{\text{i}}=0$ sets the minimum learning rate, ${\text{η}}_{\text{max}}^{\text{i}}=0.01$ establishes the maximum learning rate, and ${\text{T}}_{\text{i}}=50$ denotes the number of epochs in the iterative training process. This learning rate schedule employs a gradual diminution strategy, enabling precise model refinement throughout the training phase.

For further refinement of the training approach and to increase predictive accuracy, we utilized stochastic gradient descent as the optimization technique. Additionally, softmax cross-entropy served as the loss function, aiding in calculating the probability distribution over the intended target classes.

### Multi-instance learning for WSI integration

Upon completing the training of our deep learning model, we directed our efforts towards predicting labels and corresponding probabilities for individual patches. Subsequently, these probabilities were aggregated through a classifier to formulate predictions at the WSI level. In our study, we employed the densenet121 model to predict labels and obtain corresponding probabilities for each patch, denoted as ${\text{Patch}}_{\text{prob}}$ and ${\text{Patch}}_{\text{pred}}$ , respectively. The prediction probabilities were precisely rounded to two decimal places.

In our study, we developed two machine learning strategies for integrating patch-level probabilities. Firstly, employing histogram feature aggregation for the Probability Label Heatmap (PLH), we categorized each unique numerical value as a “bin,” monitoring the occurrence of data types within these bins. We specifically tallied the frequencies of ${\text{Patch}}_{\text{prob}}$ and ${\text{Patch}}_{\text{pred}}$ in each bin and applied min-max normalization across all features. This process culminated in the generation of ${\text{Histo}}_{\text{prob}}$ and ${\text{Histo}}_{\text{pred}}$ , enhancing data interpretability. Secondly, we implemented the Bag of Words (BoW) feature aggregation method, initiating with a comprehensive dictionary comprising unique dataset elements. Each patch was vectorized according to the presence of these elements, with further refinement via term frequency-inverse document frequency (TF-IDF) transformation, emphasizing the significance of unique, informative features. This approach yielded a BoW feature representation for each patch, effectively encapsulating feature presence and relevance. The final BoW features, denoted as ${\text{BoW}}_{\text{prob}}$ and ${\text{BoW}}_{\text{pred}}$ offered a comprehensive, weighted overview, priming them for advanced analytical applications.

In the final phase of our feature fusion approach, based on multi-instance learning, we integrated previously derived features: ${\text{Histo}}_{\text{prob}}$ , ${\text{Histo}}_{\text{pred}}$ , ${\text{Bow}}_{\text{prob}}$ , and ${\text{Bow}}_{\text{pred}}$ . To accomplish this integration, we employed a feature concatenation method symbolized by ⊕ , effectively merging these distinct feature sets into a single, comprehensive feature vector. The specific formula for this concatenation is as follows:


$$feature_{fusion}=Histo_{prob}⊕Histo_{pred}⊕Bow_{prob}⊕Bow_{pred}$$

### Pathomics signature

In our study, we developed a nuanced pathomics signature by integrating patch-level predictions, probability histograms, and TF-IDF features to create individualized patient profiles. To refine feature selection, we employed the Pearson correlation coefficient, retaining only one feature from each pair with a correlation exceeding 0.9. The model integrates a diverse array of machine learning methodologies, encompassing Logistic Regression (LR), Support Vector Machine (SVM), Random Forest, LightGBM, ExtraTrees, and XGBoost. Together, these techniques form what is termed the pathomics signature.

### Model evaluation and statistical analysis

Model accuracy was evaluated through receiver operating characteristic (ROC) curves. Statistical analyses, comprising independent sample t-tests for continuous variables and χ² tests for discrete variables, were performed to evaluate differences in patients’ clinical characteristics. Univariate and multivariate logistic regression analyses were utilized to examine clinical characteristics, retaining those with *P*-values< 0.05 in the combined model for further use. For practical clinical application, we integrated significant clinical characteristics with the pathomics signature into a combined model, which is visualized through a nomogram for ease of interpretation.

All selected patients were regularly followed up through outpatient visits and telephone check-ins. During the follow-up period, they underwent routine physical examinations and chest-enhanced computed tomography (CT) scans, with additional tests such as positron emission tomography-computed tomography (PET-CT), ultrasound, bronchoscopy, magnetic resonance imaging (MRI), or whole-body bone scans as necessary. For patients with more than one month since the last recorded entry in the case system, we conducted telephone follow-ups to assess their condition and survival status. The last follow-up for all patients was conducted on August 18, 2024, with a median follow-up time of 21 months (range: 3-44 months). In our study, DFS was defined as the interval from the date of curative lung cancer resection to the first occurrence of recurrence, metastasis, death from any cause, or the last follow-up. OS was defined as the time from the initiation of treatment to death from any cause or the last follow-up. Kaplan-Meier analysis was used to estimate DFS and OS, and comparisons between groups were performed using the log-rank test.

The deep learning models in this study were trained on robust hardware, including an Intel i9-14900k CPU, 64GB of RAM, and an NVIDIA RTX 4090 GPU. For our analysis, we employed a blend of software tools alongside custom scripts to achieve precise and efficient processing. Medical image segmentation and processing were facilitated using ITK-SNAP v3.8.0. Our computational work, spanning from modeling to data analysis, was primarily executed in Python v3.7.12, leveraging essential libraries such as PyTorch v1.8.0 for deep learning algorithms, scikit-learn v1.0.2 for machine learning.

## Results

### Patients data and clinical features

**Table 1** summarizes the baseline characteristics of our study cohort. Notably, the MPR rate was 63.4%(118/186). The cohort predominantly consisted of male patients, representing 89.2%(166/186), with the majority undergoing 2 to 3 cycles of neoadjuvant therapy, which accounted for 89.8%(167/186). Squamous cell carcinoma emerged as the leading histological type, comprising 72.6%(135/186) of cases, and most patients were classified under clinical TNM stage III (136/186, 73.1%). Through detailed univariate and multivariable analysis of clinical features, histology was identified as an independent prognostic factor for MPR, showing statistical significance with a *P*-value below 0.05, as illustrated in **Table 2**.

**Table 1:** Baseline characteristics of all cohorts.

Characteristics	Training cohort(n=105)			Validation cohort(n=45)			Test cohort(n=36)		
	Non-MPR (n=47)	MPR (n=58)	*P* value	Non-MPR (n=14)	MPR (n=31)	*P* value	Non-MPR (n=7)	MPR (n=29)	*P* value
Age			0.642			0.421			1.0
≥60	15(31.91)	15(25.86)		7(50.00)	10(32.26)		2(28.57)	9(31.03)	
<60	32(68.09)	43(74.14)		7(50.00)	21(67.74)		5(71.43)	20(68.97)	
Gender			0.099			0.547			0.838
Female	8(17.02)	3(5.17)		1(7.14)	6(19.35)		1(14.29)	1(3.45)	
Male	39(82.98)	55(94.83)		13(92.86)	25(80.65)		6(85.71)	28(96.55)	
Smoking status			0.339			0.948			1.0
No	18(38.30)	16(27.59)		5(35.71)	13(41.94)		1(14.29)	4(13.79)	
Yes	29(61.70)	42(72.41)		9(64.29)	18(58.06)		6(85.71)	25(86.21)	
Alcohol status			0.119			1.0			1.0
No	36(76.60)	35(60.34)		10(71.43)	22(70.97)		4(57.14)	19(65.52)	
Yes	11(23.40)	23(39.66)		4(28.57)	9(29.03)		3(42.86)	10(34.48)	
Histology			0.004			0.549			0.049
Adenocarcinoma	24(51.06)	13(22.41)		3(21.43)	3(9.68)		4(57.14)	4(13.79)	
SCC	23(48.94)	45(77.59)		11(78.57)	28(90.32)		3(42.86)	25(86.21)	
Surgical approach			0.383			1.0			0.808
Thoracotomy	30(63.83)	31(53.45)		8(57.14)	18(58.06)		1(14.29)	8(27.59)	
VATS	17(36.17)	27(46.55)		6(42.86)	13(41.94)		6(85.71)	21(72.41)	
Neoadjuvant therapy cycle			0.583			0.053			0.634
1	1(2.13)	0		1(7.14)	0		1(14.29)	1(3.45)	
2	24(51.06)	35(60.34)		5(35.71)	20(64.52)		3(42.86)	12(41.38)	
3	17(36.17)	18(31.03)		8(57.14)	8(25.81)		3(42.86)	14(48.28)	
4	5(10.64)	5(8.62)		0	3(9.68)		0	2(6.90)	
Clinical T stage			0.839			0.819			0.591
1	3(6.38)	2(3.45)		2(14.29)	2(6.45)		2(28.57)	5(17.24)	
2	22(46.81)	26(44.83)		5(35.71)	12(38.71)		2(28.57)	16(55.17)	
3	11(23.40)	17(29.31)		4(28.57)	8(25.81)		2(28.57)	4(13.79)	
4	11(23.40)	13(22.41)		3(21.43)	9(29.03)		1(14.29)	4(13.79)	
Clinical N stage			0.277			0.19			0.447
0	10(21.28)	9(15.52)		2(14.29)	13(41.94)		0	5(17.24)	
1	9(19.15)	19(32.76)		4(28.57)	6(19.35)		2(28.57)	9(31.03)	
2	28(59.57)	30(51.72)		8(57.14)	12(38.71)		5(71.43)	15(51.72)	
Clinical TNM stage			0.13			0.571			0.285
I	2(4.26)	1(1.72)		0	1(3.23)		0	0	
II	7(14.89)	18(31.03)		3(21.43)	10(32.26)		0	8(27.59)	
III	38(80.85)	39(67.24)		11(78.57)	20(64.52)		7(100.00)	21(72.41)	
Surgical procedure			0.242			0.445			0.087
Bilobectomy	6(12.77)	5(8.62)		3(21.43)	3(9.68)		0	3(10.34)	
Lobectomy	36(76.60)	51(87.93)		10(71.43)	27(87.10)		6(85.71)	26(89.66)	
Pneumonectomy	5(10.64)	2(3.45)		1(7.14)	1(3.23)		1(14.29)	0	

MPR, major pathologic response; Non-MPR, non-major pathologic response; SCC, Squamous cell carcinoma; VATS, video-assisted thoracic surgery.

**Table 2:** Univariable and multivariable analysis for predicting major pathological response in non-small cell lung cancer after neoadjuvant chemoimmunotherapy.

Variables	Univariate Analysis		Multivariate Analysis	
	OR(95% CI)	*P* value	OR(95% CI)	*P* value
Age (<60)	1.255 (0.661, 2.380)	0.487		
Smoking status (No)	1.455 (0.766, 2.760)	0.252		
Alcohol status (No)	1.485 (0.769, 2.868)	0.239		
Gender (Female)	1.912 (0.752, 4.862)	0.173		
Histology (Adenocarcinoma)	3.784 (1.929, 7.423)	<0.001	3.784(1.929,7.423)	<0.001
Surgical approach (Thoracotomy)	1.378 (0.755, 2.517)	0.297		
Neoadjuvant therapy cycle				
1	Reference			
2	6.281 (0.628, 62.780)	0.118		
3	4.556 (0.450, 46.113)	0.199		
4	6.000 (0.490, 73.455)	0.161		
Clinical T stage				
1	Reference			
2	1.117 (0.369, 3.384)	0.845		
3	1.024 (0.316, 3.318)	0.969		
4	1.040 (0.315, 3.436)	0.949		
Clinical N stage				
0	Reference			
1	1.007 (0.405, 2.507)	0.987		
2	0.644 (0.292, 1.420)	0.276		
Clinical TNM stage				
I	Reference			
II	3.600 (0.449, 28.858)	0.228		
III	1.473 (0.201, 10.770)	0.703		
Surgical procedure				
Bilobectomy	Reference			
Lobectomy	1.684 (0.656, 4.322)	0.278		
Pneumonectomy	0.351 (0.070, 1.761)	0.203		

OR, odds ratio; CI, confidence interval; SCC, squamous cell carcinoma; VATS, video-assisted thoracic surgery.

The performance of the clinical model, assessed using the area under the curve (AUC) metric, revealed distinct levels of discrimination capability across various machine learning algorithms and datasets. LR exhibited modest effectiveness in the training set (AUC = 0.735), but its performance significantly declined in the validation set (AUC = 0.484), highlighting a notable reduction in its discriminative power. The SVM algorithm demonstrated superior discrimination in the training set (AUC = 0.871), though it achieved only moderate results in the validation set (AUC = 0.585). Both Random Forest and ExtraTrees algorithms displayed commendable results in the training set (AUC = 0.799 and 0.810, respectively), yet their efficacy was moderate to modest in the validation and test sets. XGBoost demonstrated consistent performance with good results in both the training (AUC = 0.722) and test sets (AUC = 0.714), but it exhibited suboptimal performance in the validation set (AUC = 0.492). LightGBM presented moderate performance across all datasets, with AUC scores of 0.674, 0.563, and 0.667 for the training, validation, and test sets, respectively (**Supplementary Figure 1**).

### Pathomics signature

**Figure 3:**
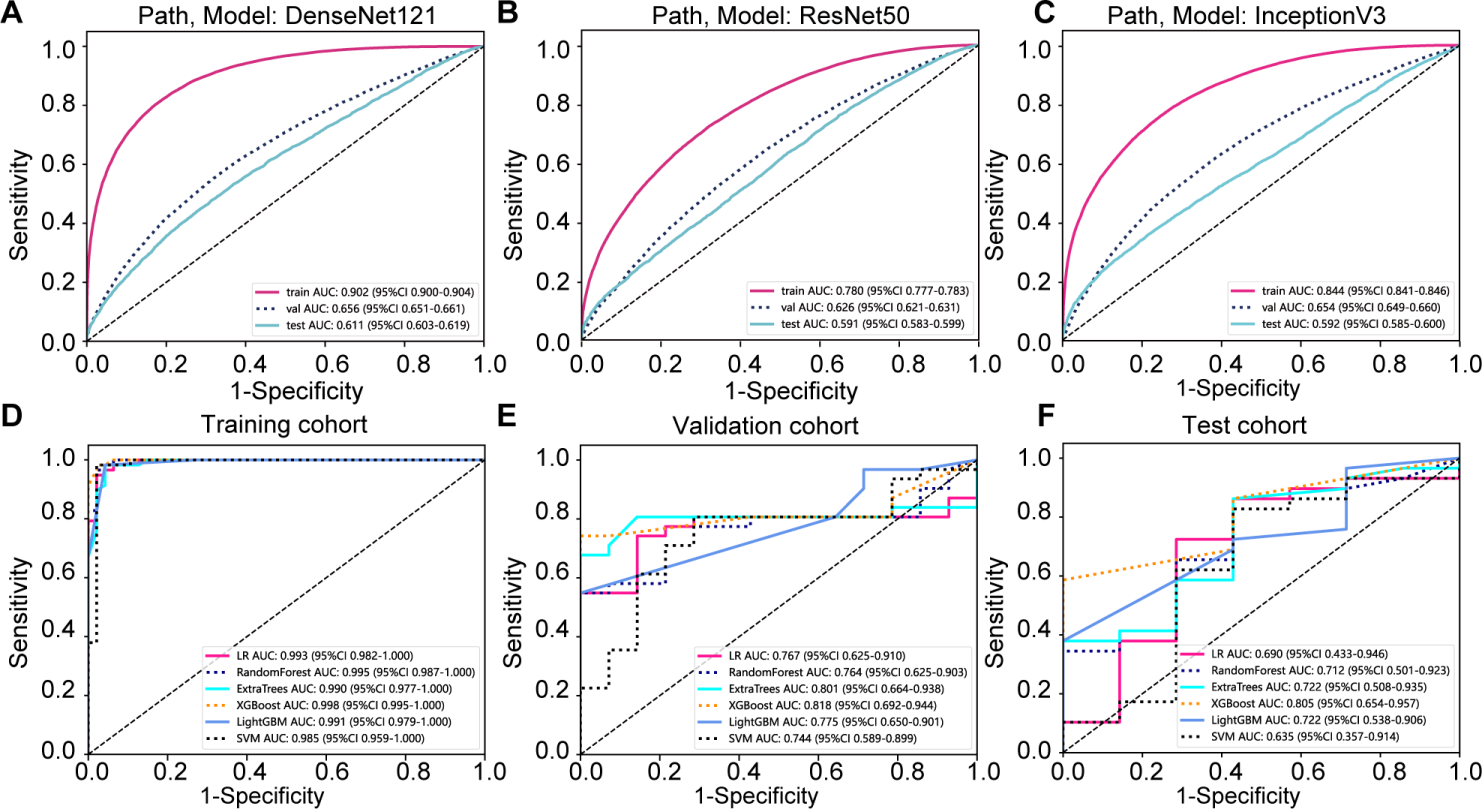
Prediction model evaluation. **(A)** Patch-level area under the curve (AUC) for the DenseNet121 prediction model across cohorts; **(B)** Patch-level AUC for the Resnet50 prediction model; **(C)** Patch-level AUC for the Inception v3 prediction model; **(D)** whole slide image (WSI)-level AUC for the prediction model in the training cohort; **(E)** WSI-level AUC for the prediction model in the validation cohort; **(F)** WSI-level AUC for the prediction model in the testing cohort.

Our pathology models’ ability to discern regional features was rigorously evaluated using ROC curves at the patch level. DenseNet121, among the models assessed, distinguished itself in the validation set, achieving an AUC of 0.656 (95% CI: 0.651-0.661), thereby surpassing both ResNet50 (AUC = 0.626, 95% CI: 0.621-0.631) and Inception V3 (AUC = 0.654, 95% CI: 0.649-0.660). Additionally, DenseNet121 demonstrated commendable generalization with an AUC of 0.611 (95% CI: 0.603-0.619) in the external test set. The comparative analysis of these models is illustrated in **Figures 3A–C**.

To further evaluate our model’s effectiveness, we visually inspected the amalgamation of patch-level data into WSIs. Among the machine learning techniques assessed, XGBoost outperformed others, delivering the highest AUC scores in the training and testing phases. With an AUC of 0.998 in the training phase, XGBoost demonstrated exemplary discriminative prowess. In the validation phase, it achieved a robust AUC of 0.818 and maintained a strong AUC of 0.805 in the testing phase, indicating reliable performance. XGBoost’s consistent AUC superiority reveals its remarkable capacity for class differentiation and generalization to unseen data (**Figures 3D–F**).

**Figure 4:**
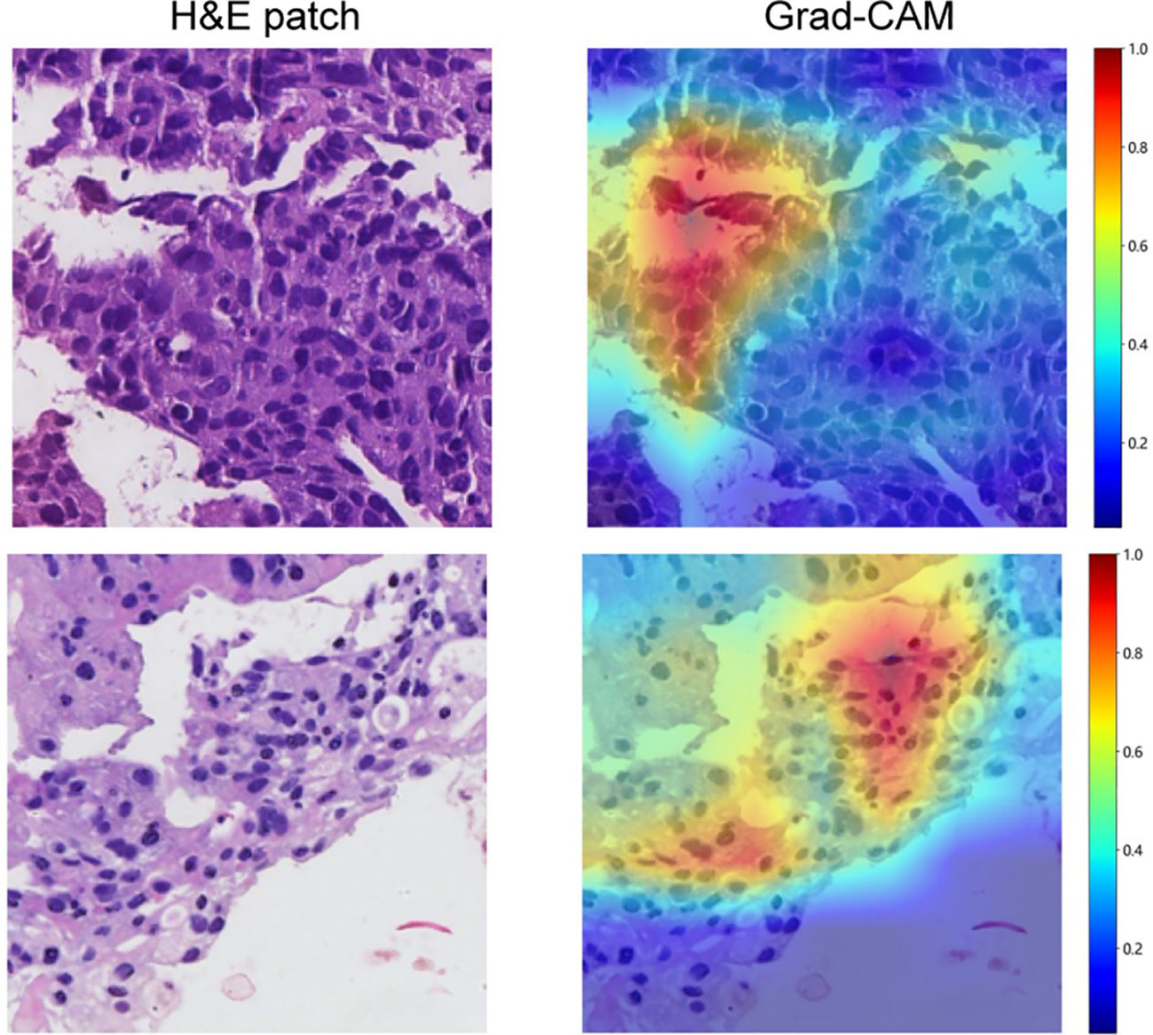
Use of Grad-CAM to illustrate activation in the final convolutional layer of the prediction model.

**Figure 5:**
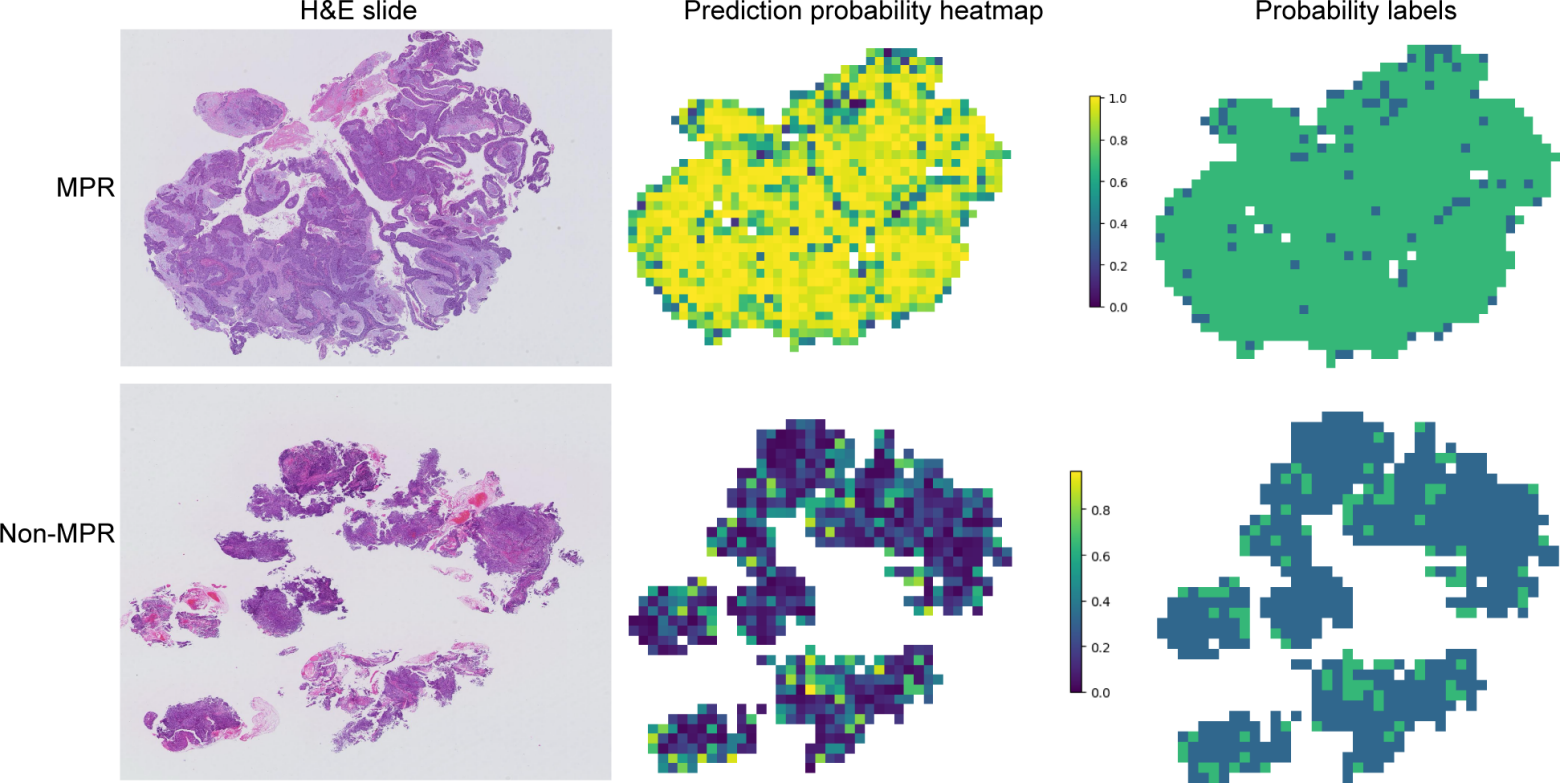
Probability and prediction heatmap of the prediction model. This image displays the whole slide image (WSI)-level hematoxylin and eosin slide (left), a heatmap of the prediction probabilities for each patch (middle), and the result prediction map for the WSI (right). Major pathological response (MPR) is primarily predicted with a probability label of 1, whereas non-major pathological response (Non-MPR) is predominantly predicted with a probability label of 0.

Gradient-weighted class activation mapping (Grad-CAM) generates visual maps by tracing gradients in the network’s final convolutional layer, preserving key spatial details relevant to the classification task, details that are often lost in fully connected layers. This technique seamlessly fits into existing neural architectures without necessitating any model modifications or retraining. **Figure 4** demonstrates this, by providing a clear depiction of the last convolutional layer’s contribution in the model’s predictive response, enhancing interpretability of the model’s decision-making. Predictive label and probability heatmaps were obtained to assist in the evaluation. As depicted in **Figure 5**, the prediction heatmap vividly showcases our pathological model’s high accuracy when assessing regional tiles. The results indicate that feature modeling has been notably improved following aggregation via the BoW and PLH processes. This signifies the efficacy of our feature aggregation methodology.

### Model fusion and performance

**Figure 6:**
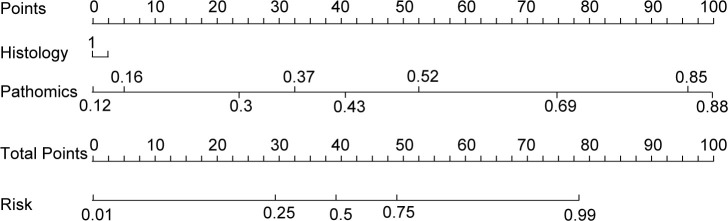
Clinical nomogram to predict major pathological response in non-small cell lung cancer patients post-neoadjuvant chemoimmunotherapy.

**Figure 7:**
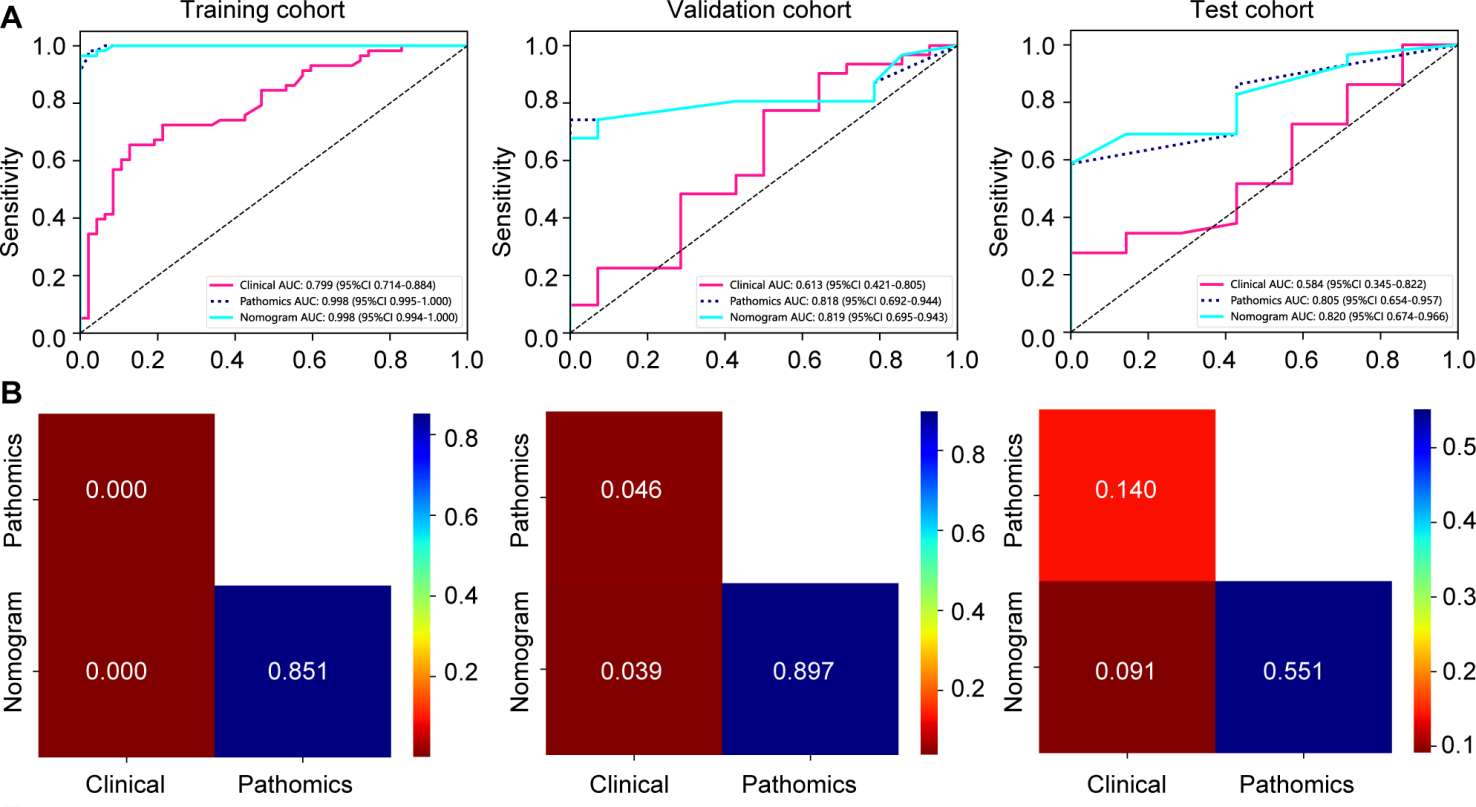
Assessment of model efficacy in forecasting major pathological responses to neoadjuvant chemoimmunotherapy in training, validation, and test cohorts. **(A)** receiver operating characteristic curves depicting prediction accuracy of signatures; **(B)** DeLong test comparisons among various signatures.

The nomogram, an integrative tool combining clinical and pathomics information, is effectively illustrated in **Figure 6**. The assessment of AUC scores for clinical, pathomics, and nomogram signatures indicates that the nomogram consistently secures marginally higher AUC values than the pathomics signature on its own, observed in both validation and test datasets. Notably, the nomogram records an AUC of 0.819 in the validation group and 0.820 in the test group, reflecting an effective amalgamation of features for refined discriminative accuracy. The pathomics signature alone exhibits formidable discriminative strength across all datasets, with an almost perfect AUC of 0.998 in training. The clinical signature, however, reveals a descending trajectory in discriminative capacity, with an AUC drop from 0.799 in training to 0.613 in validation and further to 0.584 in testing, thereby emphasizing the incremental benefit of pathomics feature integration in enhancing model efficacy (**Supplementary Table 1**, **Figure 7A**). Utilizing the DeLong test for statistical comparison, the nomogram, which synthesizes clinical and pathomics attributes, demonstrated augmented predictive superiority. The performance elevation of the nomogram over the clinical-only model was statistically significant, registering a *P*-value less than 0.05, hence confirming the added value of integrating pathomics insights into clinical predictions (**Figure 7B**).

### Clinical outcomes

**Figure 8:**
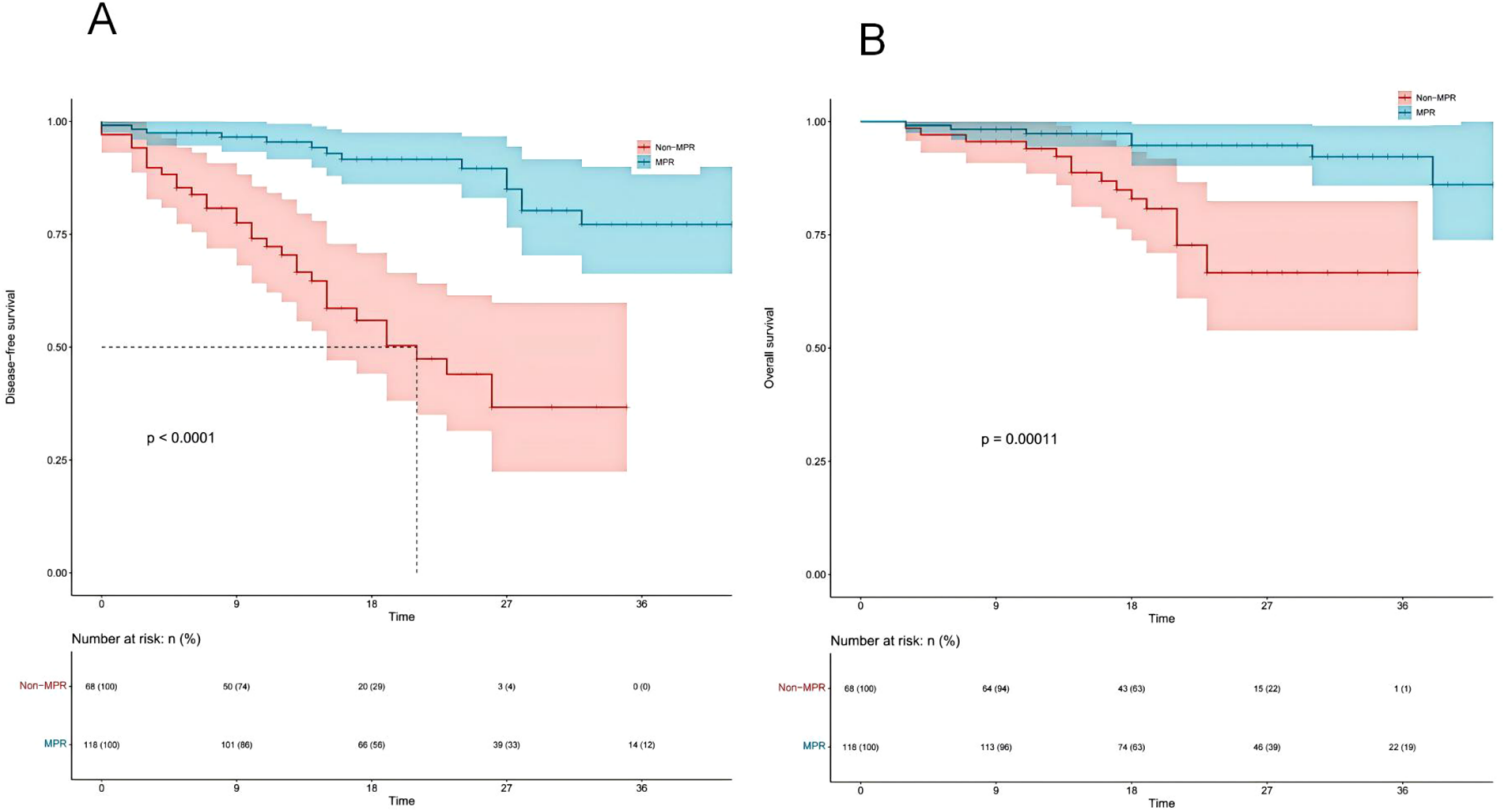
Kaplan-Meier survival analysis of disease-free survival (DFS) **(A)** and overall survival (OS) **(B)** between major pathological response (MPR) and non-major pathological response (Non-MPR) groups.

**Figures 8A, B** present the DFS and OS curves for patients treated with NICT, comparing the MPR group to the Non-MPR group. The analysis reveals that the MPR group exhibited significantly improved DFS and OS outcomes, with statistically significant differences between the two cohorts.

## Discussion

In this study, our objective was to develop an accurate predictive model for MPR in NSCLC patients undergoing NICT. By integrating machine learning analyses of clinical data, we established a clinical signature grounded in machine learning principles. Moreover, we assessed the predictive value of pathomics data on MPR outcomes. Leveraging a weakly supervised deep learning framework trained on WSIs with multi-instance aggregation, we achieved precise predictions of MPR at the patient level, culminating in the establishment of a pathomics signature. The pinnacle of our study involved merging the derived clinical features with the pathomics signature into a unified nomogram, crafted for extensive interpretability and detailed examination, offering a methodology for MPR prediction in NSCLC patients receiving NICT. This integrated approach represents a significant fusion of clinical insights with advanced machine learning techniques and, to our knowledge, it pioneers the use of WSI for the first-time prediction of MPR in NSCLC patients treated with NICT, setting a new benchmark in the field.

MPR is gaining recognition as a pivotal prognostic marker in resectable NSCLC, particularly when considering the context of NICT. The capability of MPR to accurately mirror the tumor’s response to therapeutic interventions is essential for predicting patient outcomes effectively. Research demonstrates MPR’s link to improved long-term OS among NSCLC patients who receive neoadjuvant chemotherapy, underscoring its significance as both a surrogate endpoint for survival and a critical measure for evaluating neoadjuvant therapy in clinical trials (28). Additionally, comprehensive studies exploring the prognostic relevance of MPR in NSCLC patients undergoing NICT have found a strong correlation with enhanced DFS and OS, supporting the use of MPR as a surrogate marker for survival outcomes in the evaluation of NICT’s effectiveness (2, 4, 6, 29, 30). Our research, in conjunction with these findings, accentuates the crucial role of MPR in assessing the success of neoadjuvant treatment approaches in NSCLC, thereby validating our decision to use MPR as a predictor of NICT’s efficacy in this clinical setting.

In the field of deep learning model development, access to large datasets and high-quality annotations is crucial for training high-performance models. However, the high resolution of WSI presents significant challenges for detailed annotation. Consequently, researchers have developed a new training method using limited annotations, known as weak supervision (31, 32). In the realm of WSI classification under weak supervision, a significant portion of research has predominantly concentrated on employing multiple instance learning (MIL) techniques (33–36). The MIL approach identifies the relative importance of each image patch for model prediction by analyzing histopathological images, allowing the model to autonomously learn to recognize morphological features of diseases without the need for manual annotations. In this research, we applied a weakly supervised learning framework using MIL on pre-treatment WSIs to forecast MPR in NSCLC patients post-NICT, achieving an AUC of 0.998 in training, and demonstrating robust performance with AUCs of 0.818 in validation and 0.805 in testing phases. Additionally, we enhanced our model’s interpretability in decision-making by utilizing GradCAM localization mapping, which facilitated the evaluation through predictive labels and probability heatmaps. GradCAM uniquely enables target localization in models trained using only image labels by incorporating guided backpropagation, precisely determining pixel-level importance in predictive areas, thus offering significant benefits for applications like cancer subtype classification (37–39).

This study has several limitations, including a small sample size and reliance on a retrospective cohort, which may affect the generalizability of our findings. To validate our results and strengthen the conclusions drawn, future research with a larger sample size and a prospective design is essential. Furthermore, the developed model focuses on pathological images and clinical features without incorporating conventional imaging data, such as CT scans, or molecular information like genetic and protein expressions. Acknowledging the dynamic nature of AI models, future iterations will aim to incorporate multidimensional patient data to enhance the performance of model predictions.

Moving forward, our research will focus on several key areas. First, we plan to conduct prospective studies to validate our findings and evaluate the model’s applicability across diverse populations and clinical settings. Additionally, we aim to develop advanced visualization and interpretation tools to improve model transparency and facilitate its use by clinicians in decision-making processes. Finally, we will explore strategies for integrating the predictive model into existing clinical workflows, with an emphasis on feasibility, usability, and acceptance in real-world clinical environments.

## Conclusion

The utilization of weakly supervised deep learning for analyzing WSIs provides a potent predictive tool for MPR in NSCLC patients undergoing NICT. By enhancing treatment precision, this model promises not only to improve patient outcomes but also to refine therapeutic strategies. Future work will aim to incorporate extensive multimodal data, further improving the predictive accuracy and robustness of our models.

## Data Availability

The raw data supporting the conclusions of this article will be made available by the authors, without undue reservation.
