# Supplementary material for: Whole slide image-based weakly supervised deep learning for predicting major pathological response in non-small cell lung cancer following neoadjuvant chemoimmunotherapy: a multicenter, retrospective, cohort study
Source: Front Immunol. 2024 Sep 20;15:1453232. doi: 10.3389/fimmu.2024.1453232 (PMC11449764; doi:10.3389/fimmu.2024.1453232)
Supplement: Supplementary file 2 [file DataSheet2.docx]

Supplementary Material

1. **Supplementary Methods**

In this study, the primary preoperative chemotherapy regimen for squamous cell carcinoma patients comprised intravenous administration of paclitaxel-like and platinum-based drugs. Conversely, patients with adenocarcinoma were treated with intravenous pemetrexed in combination with platinum-based drugs. The platinum-based chemotherapies included either carboplatin (administered at an area under the curve of 5) or cisplatin (25 mg/m² on days 1–3). Paclitaxel regimens varied, including paclitaxel (135–175 mg/m²) and albumin-bound paclitaxel (260 mg/m²), supplemented by pemetrexed at a dose of 500 mg/m². Preoperative immunotherapy was administered using programmed cell death protein 1 (PD-1) inhibitors: tislelizumab, pembrolizumab, camrelizumab, and sintilimab (each at a dose of 200 mg), nivolumab (360 mg), and toripalimab (240 mg). Typically, patients received 1-3 doses of these regimens every three weeks, with an average of two cycles.

Patients underwent radical surgical resection of lung cancer under general anesthesia, 4 to 6 weeks following their last neoadjuvant treatment. Surgical approaches included video-assisted thoracoscopic surgery (VATS) and thoracotomy. Depending on individual patient factors and tumor characteristics, the extent of resection varied among lobectomy, sleeve resection, and total unilateral pneumonectomy.

1. **Supplementary Figures and Tables**

**Supplementary Figure 1.** Comparative receiver operating characteristic curve analysis for the clinical signature across training, validation, and test cohorts using machine learning models.


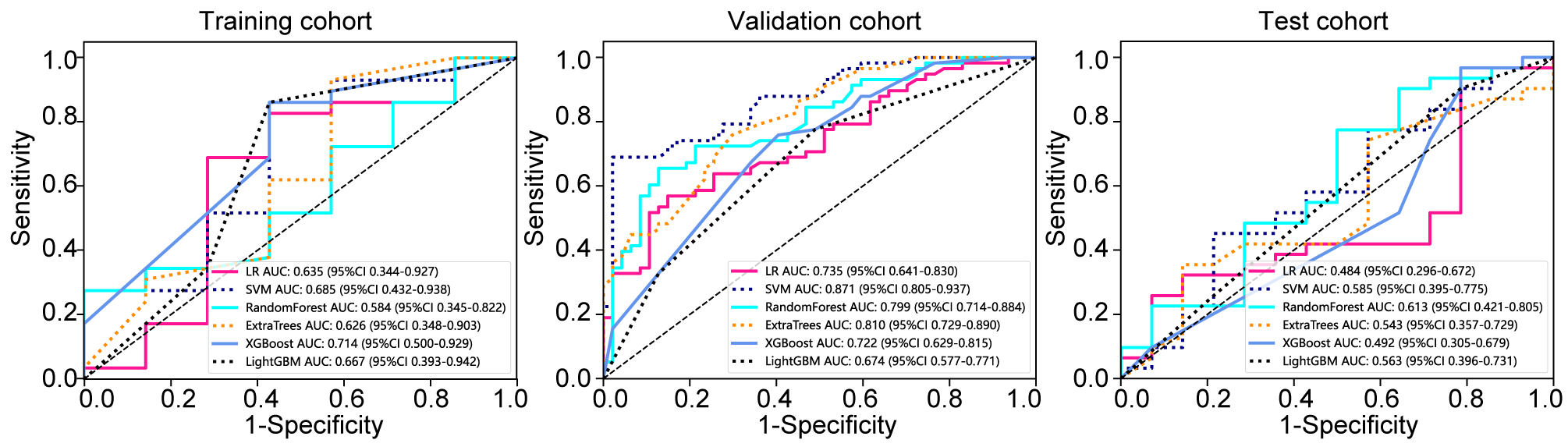


**Supplementary Table 1**. Comparative performance metrics of different models for predicting major pathological response in non-small cell lung cancer patients post-neoadjuvant chemoimmunotherapy.

| Cohort | Signature | AUC | 95% CI | Accuracy | Sensitivity | Specificity |
| --- | --- | --- | --- | --- | --- | --- |
| training | Clinical | 0.799 | 0.7144 - 0.8835 | 0.743 | 0.638 | 0.872 |
|  | Pathomics | 0.998 | 0.9949 - 1.0000 | 0.971 | 0.966 | 0.979 |
|  | Nomogram | 0.998 | 0.9944 - 1.0000 | 0.952 | 0.914 | 1.000 |
| validation | Clinical | 0.613 | 0.4209 - 0.8049 | 0.711 | 0.871 | 0.357 |
|  | Pathomics | 0.818 | 0.6924 - 0.9435 | 0.689 | 0.806 | 0.429 |
|  | Nomogram | 0.819 | 0.6950 - 0.9432 | 0.689 | 0.806 | 0.429 |
| test | Clinical | 0.584 | 0.3453 - 0.8222 | 0.750 | 0.862 | 0.286 |
|  | Pathomics | 0.805 | 0.6540 - 0.9568 | 0.694 | 0.724 | 0.571 |
|  | Nomogram | 0.820 | 0.6744 - 0.9660 | 0.694 | 0.724 | 0.571 |

AUC: area under the curve; CI: confidence interval.
